# Residues located in the primase domain of the bacteriophage T7 primase-helicase are essential for loading the hexameric complex onto DNA

**DOI:** 10.1016/j.jbc.2022.101996

**Published:** 2022-04-30

**Authors:** Alfredo J. Hernandez, Seung-Joo Lee, Noah J. Thompson, Jack D. Griffith, Charles C. Richardson

**Affiliations:** 1 Department of Biological Chemistry and Molecular Pharmacology, Harvard Medical School, Boston, Massachusetts, USA; 2 Department of Microbiology and Immunology, Lineberger Comprehensive Cancer Center, The University of North Carolina-Chapel Hill, Chapel Hill, North Carolina, USA

**Keywords:** DNA helicase, enzyme mutation, protein–DNA interaction, bacteriophage, viral protein, multifunctional enzyme, mass spectrometry, site-directed mutagenesis, dGMP, deoxy-GMP, dGTP, deoxy-GTP, dNTP, deoxy-NTP, dTTP, deoxy-TTP, RPD, RNA polymerase domain, TOPRIM, topoisomerase-primase, ZBD, zinc-binding domain

## Abstract

The T7 primase-helicase plays a pivotal role in the replication of T7 DNA. Using affinity isolation of peptide–nucleic acid crosslinks and mass spectrometry, we identify protein regions in the primase-helicase and T7 DNA polymerase that form contacts with the RNA primer and DNA template. The contacts between nucleic acids and the primase domain of the primase-helicase are centered in the RNA polymerase subdomain of the primase domain, in a cleft between the N-terminal subdomain and the topoisomerase-primase fold. We demonstrate that residues along a beta sheet in the N-terminal subdomain that contacts the RNA primer are essential for phage growth and primase activity *in vitro*. Surprisingly, we found mutations in the primase domain that had a dramatic effect on the helicase. Substitution of a residue conserved in other DnaG-like enzymes, R84A, abrogates both primase and helicase enzymatic activities of the T7 primase-helicase. Alterations in this residue also decrease binding of the primase-helicase to ssDNA. However, mass photometry measurements show that these mutations do not interfere with the ability of the protein to form the active hexamer.

The molecular processes involved in replicating DNA are complex, requiring the concerted action of multiple proteins (1, 2). The replisome consists of the dynamic multiprotein assembly responsible for DNA replication. Despite vast differences in the number of components, the essential biochemical steps performed by the replisome are conserved throughout evolution (3, 4).

Bacteriophage T7 employs an economical replication system (2, 5). Only four proteins are required to reconstitute coordinated synthesis of leading and lagging strands: the phage-encoded DNA polymerase gp5, the ssDNA-binding protein gp2.5, the primase-helicase gp4, and *Escherichia coli* thioredoxin Trx (Fig. 1*A*). The bifunctional primase-helicase gp4 lies at the center of the T7 replisome, structurally and functionally (6, 7). Besides engaging DNA and the DNA polymerases responsible for synthesis of the leading and lagging strands, the enzymatic activities of gp4 are crucial for the function of the T7 replisome. The C-terminal helicase domain of gp4 belongs to the SF4 group of replicative hexameric helicases (8). It unwinds dsDNA using the energy of nucleotide hydrolysis in order to produce the templates for DNA polymerase (2, 5).

**Figure 1:**
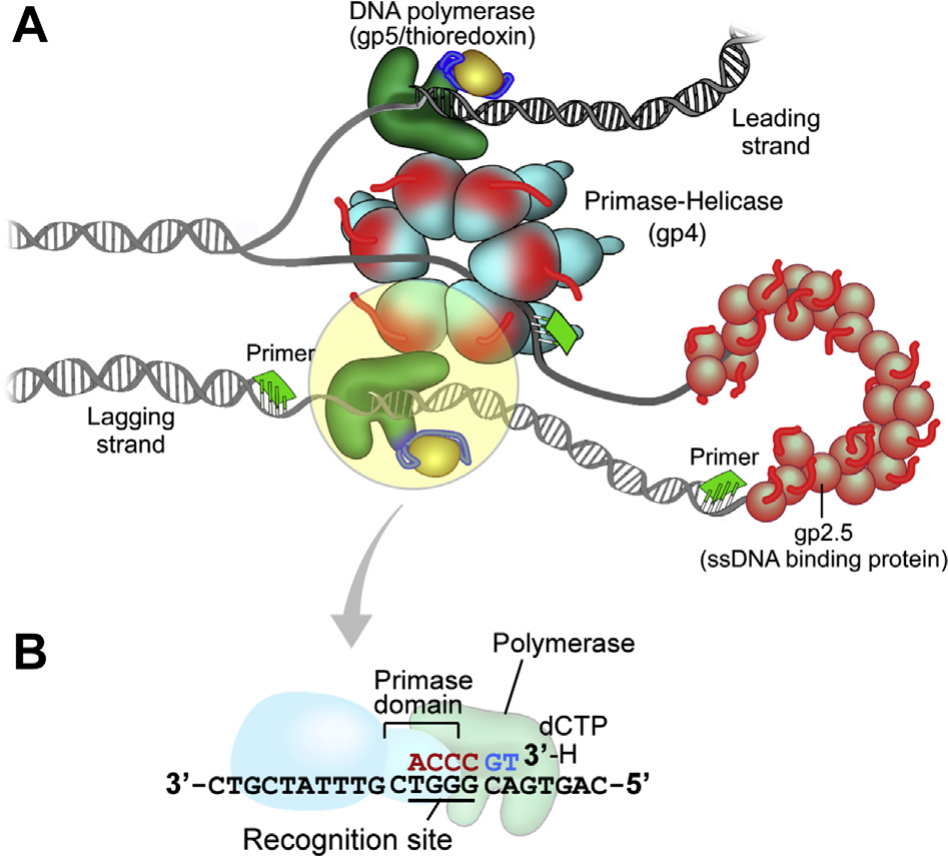
**The minimal replisome of bacteriophage T7 and isolation of a stable lagging strand priming complex.***A*, the hexameric primase-helicase (gp4) unwinds the DNA duplex to provide the template for DNA synthesis. The leading strand is continuously extended by T7 DNA polymerase (a complex of T7 gp5 and its processivity factor, *E. coli* Trx), in association with the helicase domain of gp4. The primase domain of gp4 synthesizes RNA primers on the lagging strand that are then extended by T7 DNA polymerase, leading to the formation of a replication loop containing a nascent Okazaki fragment. The T7 ssDNA-binding protein, gp2.5, stabilizes ssDNA replication intermediates, interacts with T7 DNA polymerase and gp4, and is essential for the coordination of DNA synthesis on both strands. *B*, scheme for stabilizing the T7 priming complex. We followed the procedure as described by Kato *et al.* (21). The primase domain of gp4 mediates the template-directed extension of an RNA primer by T7 DNA polymerase. The RNA primer is extended by addition of a deoxyguanosine monophosphate and a chain-terminating dideoxy-TMP. The presence of the next nucleotide encoded by the template, dCTP in this case, stabilizes the complex between T7 DNA polymerase, gp4, template and partially extended, dideoxy-terminated RNA primer. dCTP, deoxy-CTP.

The N-terminal primase domain of gp4 synthesizes tetraribonucleotides that are used as primers for the discontinuous replication of the lagging strand (9). The primase domain of gp4 belongs to the bacterial DnaG family (10). Members of the DnaG family consist of an N-terminal zinc-binding domain (ZBD) involved in sequence-specific recognition of DNA, followed by an RNA polymerase domain (RPD). The RPD is composed of two parts: a conserved C-terminal topoisomerase-primase (TOPRIM) fold, containing the active site where nucleotidyl transfer occurs, and a divergent N-terminal basic subdomain, which is thought to play a role in DNA binding (11, 12). In addition to its enzymatic role in primer synthesis, the primase domain of gp4 delivers tetraribonucleotide primers to DNA polymerase and enables their extension (13).

Hexamer formation is critical for the function of gp4 (14, 15, 16). Various studies suggest that gp4 exists in equilibrium between oligomeric forms in the absence of DNA (16, 17, 18). However, only hexamers bind ssDNA efficiently and display helicase activity (16). The mechanism of gp4 binding to DNA is not fully understood. Loading of gp4 onto ssDNA does not require loading factors. It has been postulated that gp4 loads onto ssDNA through a ring opening mechanism in which one subunit from a heptamer dissociates and the hexamer encircles ssDNA. The primase domain of gp4 could serve as the initial DNA binding event, bringing the gp4 ring in close proximity to DNA (15, 16, 17).

The replication of the lagging strand requires the continual synthesis of RNA primers by gp4 and their extension by T7 DNA polymerase to form Okazaki fragments (Fig. 1*B*). The ZBD of gp4 recognizes the trinucleotide 5′-GTC-3′ in the lagging strand template to initiate the synthesis of RNA primers (19). A direct interaction between gp4 and T7 DNA polymerase is critical to allow the extension of tetraribonucleotide primers by DNA polymerase (20, 21). In the absence of this interaction, T7 DNA polymerase is unable to utilize the short RNA primers synthesized by gp4 (13, 22). The priming complex formed between gp4, T7 DNA polymerase, and primer/template consists of multiple protein–protein and protein–nucleic acid interactions, which mediate sequence-specific template recognition, oligoribonucleotide synthesis, as well as the delivery of the primer by gp4 to T7 DNA polymerase.

Here, we identify regions in gp4 and T7 DNA polymerase that directly contact RNA primers. Residues in the primer-binding region are essential for growth of T7 bacteriophage and critical for RNA synthesis and primase activity. In addition, mutations in the primase domain of gp4 have unexpected consequences on the function of the helicase domain. One residue in particular, R84, whose sequence context is conserved among DnaG-type primases, profoundly affects the activities of gp4. This mutation hinders binding of the hexamer to ssDNA, thus affecting many of the enzymatic activities of gp4.

## Results

### Identification of residues involved in primer synthesis and hand off to DNA polymerase

We sought to identify regions of the T7 primase-helicase gp4 and T7 DNA polymerase that directly contact the RNA primer during synthesis. It is likely that these regions are involved in the synthesis and hand off of RNA primers and could regulate these processes. We used formaldehyde crosslinking, coupled with affinity chromatography and mass spectrometry (23), to identify regions in gp4 and T7 DNA polymerase in contact with the primer/template. The crosslinking was carried out under conditions that favor assembly of a stable lagging-strand priming complex, consisting of T7 DNA polymerase, gp4, ssDNA template, and a dideoxy-terminated RNA primer in the presence of the next encoded deoxy-NTP (dNTP) (Fig. 1*B*) (20, 21).

The T7 lagging-strand priming complex was assembled as described, with slight modifications based on our single-turnover reaction conditions (22, 24). In one iteration, the template was biotinylated at the 3′-end, while in another, the primer was biotinylated at its 5′ end. After complex assembly, the proteins were crosslinked with formaldehyde and digested with trypsin. Oligonucleotide–peptide crosslinks were isolated using streptavidin beads and extensively washed with a buffer containing high salt to remove noncrosslinked material. After reversal of the crosslinks using heat, the samples were analyzed by mass spectrometry, and the resulting peptides mapped to gp4 or T7 DNA polymerase.

**Figure 2:**
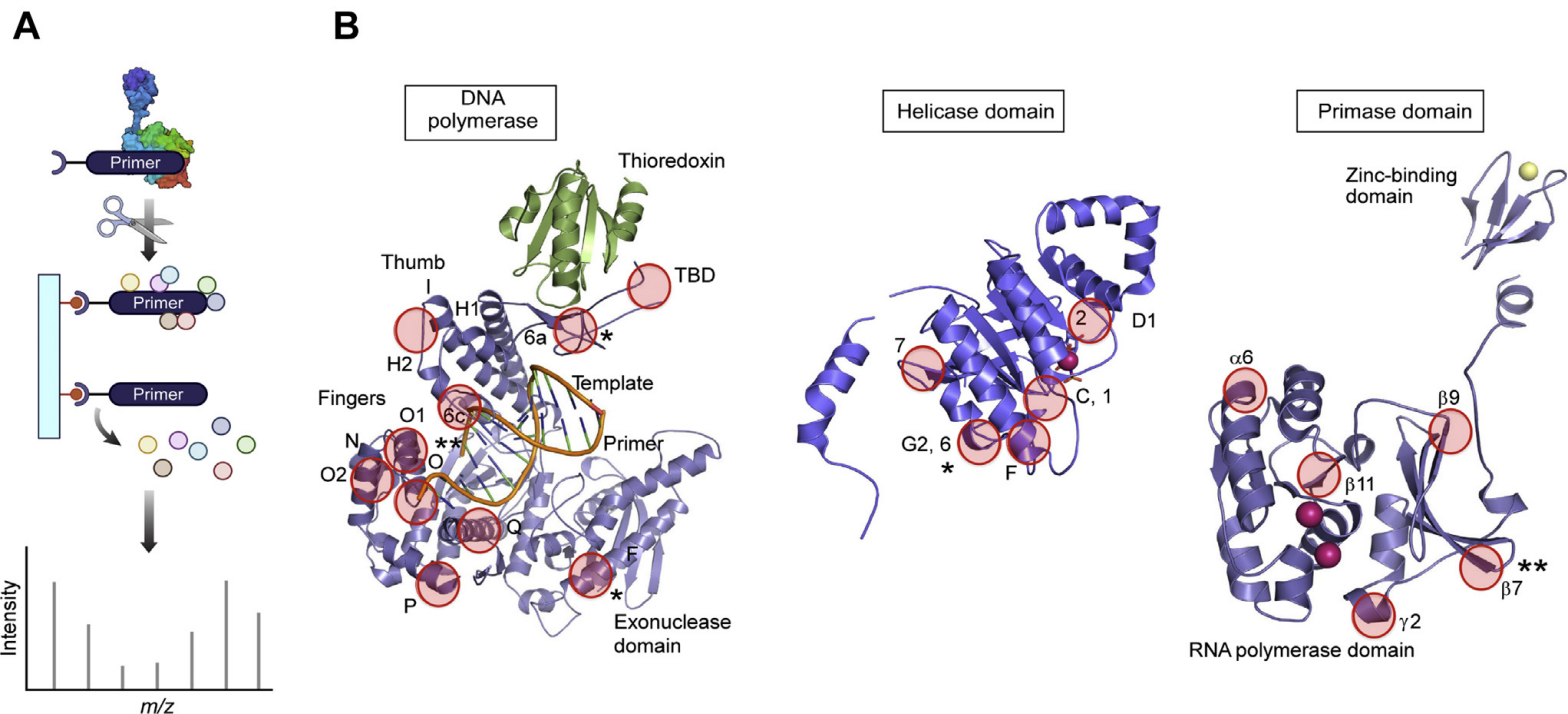
**Identification of regions of T7 DNA polymerase and gp4 in the priming complex interact with nucleic acids**. *A*, a stable complex between T7 DNA polymerase, gp4, and primer/template was assembled using either biotinylated primer or biotinylated template. Samples were crosslinked with formaldehyde and then digested with trypsin. Peptide–nucleic acid adducts were affinity purified using streptavidin magnetic beads. After reversal of crosslinks, samples were analyzed by mass spectrometry. *B*, location of peptide regions identified by crosslinking/mass spectrometry (*red circles*) in the crystal structures of T7 DNA polymerase (*left*), the helicase domain of gp4 (*middle*), and the primase domain of gp4 (*right*). ∗ indicates peptides isolated only with biotinylated template; ∗∗ indicates peptides isolated only with biotinylated primer.

The regions of DNA polymerase in close contact with nucleic acids are mostly centered on helices H1, 6c, 6d, and H2 in the thumb subdomain (residues 346–355, 360–373, and 388–394) as well as helices N, O, O1, and O2, in the finger subdomain (residues 509–518, 523–536, 537–545, and 555–566) (Fig. 2*B* and Table 1) (25). Interestingly, residues 179 to 189 of the exonuclease domain of the polymerase and 269 to 288 and 294 to 302 of the thioredoxin-binding domain crosslink to the template. In complex with Trx, the thioredoxin-binding domain is thought to form a clamp around the primer/template to aid polymerase processivity (25, 26, 27). A loop, unique to T7 DNA polymerase, residues 401 to 404, which was previously implicated in primer utilization (28) was not identified with either template nor primer, suggesting another role in the catalytic cycle of the polymerase.

**Table 1:** Peptides of gp5 and gp4 that crosslinked to either DNA or RNA identified by mass spectrometry

Peptide isolated *via* primer or template	*gp4 peptides*	*Secondary structure element/subdomain*	*Role*
	Y78-K88	78–82 unstructured/82–85 GAMMA2 310 helix/86–88 motif II	Primase motifs II and III are located in the connecting loops between a pair of helices (2/2) and a pair of strands (7/8) within this subdomain. These conserved residues face the shallow cleft separating the N-terminal subdomain from the TOPRIM fold
Primer	V101-R112	BETA7, immediately N-terminal to motif III	near K122,128, 131, 137
	T132-K144	132–135 BETA9/136–144 unstructured/motif IV @150	
	Y173-K187	175–177 BETA11, 180–191 ALPHA4	
	K214-K227	ALPHA6	
	G304-K318	307–313 BETA1 (motif H1)/318–331 ALPHAC	H1 Walker A motif, P-loop. H1, H1a, H2, and H3 form dTTP binding site. Most contacts H1 and H2.
	V337-R359	337–344 BETA2 (motif H1a), 346–357 ALPHAD1	
	K440-K450	440–456 ALPHAF of motif H3	
Template	R493-R504	492–495 ALPHAG2, 497–504 BETA6 (motif H4)	Helicase DNA-binding site
	N505-R517	505–509 unstructured/514–521 BETA7	


Both	*gp5 peptides*	*Secondary structure element/subdomain*	*Role*
Template	K179-K189	64–187, helix F exonuclease domain	
Template	G269-R288	6a(264–268) polymerase ∗unique to T7 DNA pol/TBD	
	V294-K302	unst?/TBD	Three functionally important lysines that affect phage growth and polymerase activity *in vitro*
	L346-K355	H1(339–348), 6c(355–357)∗/thumb	thumb- H1, 6c, 6d, H2
	G360-R373	6d(361–363)∗, H2(365–372)/thumb	
	E388-K394	I(376–400)/thumb	
	N509-R518	N(504–514), O(517–530)/fingers	fingers- N, O, O1, O2, P
Primer	T522-K536	O(517–530), O1(533–542/fingers	O helix stacks w/dNTP @ 3′ end primer Arg 518, Lys 522, and Tyr 526 t
	I537-K545	O1(533–542), O2(544–559)/fingers	
	F555-R566	O2(544–559), P(560–574)/fingers	
	T629-K636	Q(608–635)/palm	

Residue numbers and secondary structural features are indicated.

Regions of gp4 that contact nucleic acid map to both the helicase and primase domains (Fig. 2*B* and Table 1). Contacts between nucleic acid and the gp4 helicase domain center around the H1, H1a, and H3 motifs as well as the positively charged central cavity in the hexameric ring. The later constitutes part of motif H4, consistent with its role in template binding (29, 30). One peptide of the helicase motif H4 (aa 493–504) was isolated using biotinylated template but not with biotinylated primer.

Within the primase domain, peptides corresponding to regions in the N-terminal subdomain and the TOPRIM fold were identified; no peptides corresponding to the ZBD were identified. The N-terminal-most peptide identified is derived from the N-terminal subdomain of the RPD mapping to residues 78 to 88. This 10 residue stretch contains the γ2 3_10_ helix (12) flanked by an unstructured region to the N terminus and the conserved primase motif II at the C-terminal end. Another point of contact between primase and nucleic acid is the region comprised of residues 132 to 144, which encompasses the β9 of the N-terminal subdomain and the γ3 3_10_ helix of the TOPRIM subdomain, immediately upstream of conserved primase motif IV. Other regions of the TOPRIM fold that bind nucleic acid include residues 173 to 187 containing β11 and alpha helix α4 and residues 214 to 227, which contain alpha helix α6 (12).

Interestingly, a peptide corresponding to residues 101 to 112 of the N-terminal subdomain of the RPD was isolated using biotinylated primer. This primer-specific peptide corresponds to residues 101 to 112 of gp4, which contain the β7 immediately N-terminal to the conserved primase motif III. This region is located at a distance of 14 Å from the primase active site; however, the region sits directly opposite to W69, a residue previously found to be essential for primer delivery (31), suggesting that this region could be involved in primer binding and release (22). We focused on this region to understand its role in coordinating RNA synthesis and their transfer to DNA polymerase.

### Residues involved in primer synthesis and hand off are essential for phage growth

**Figure 3:**
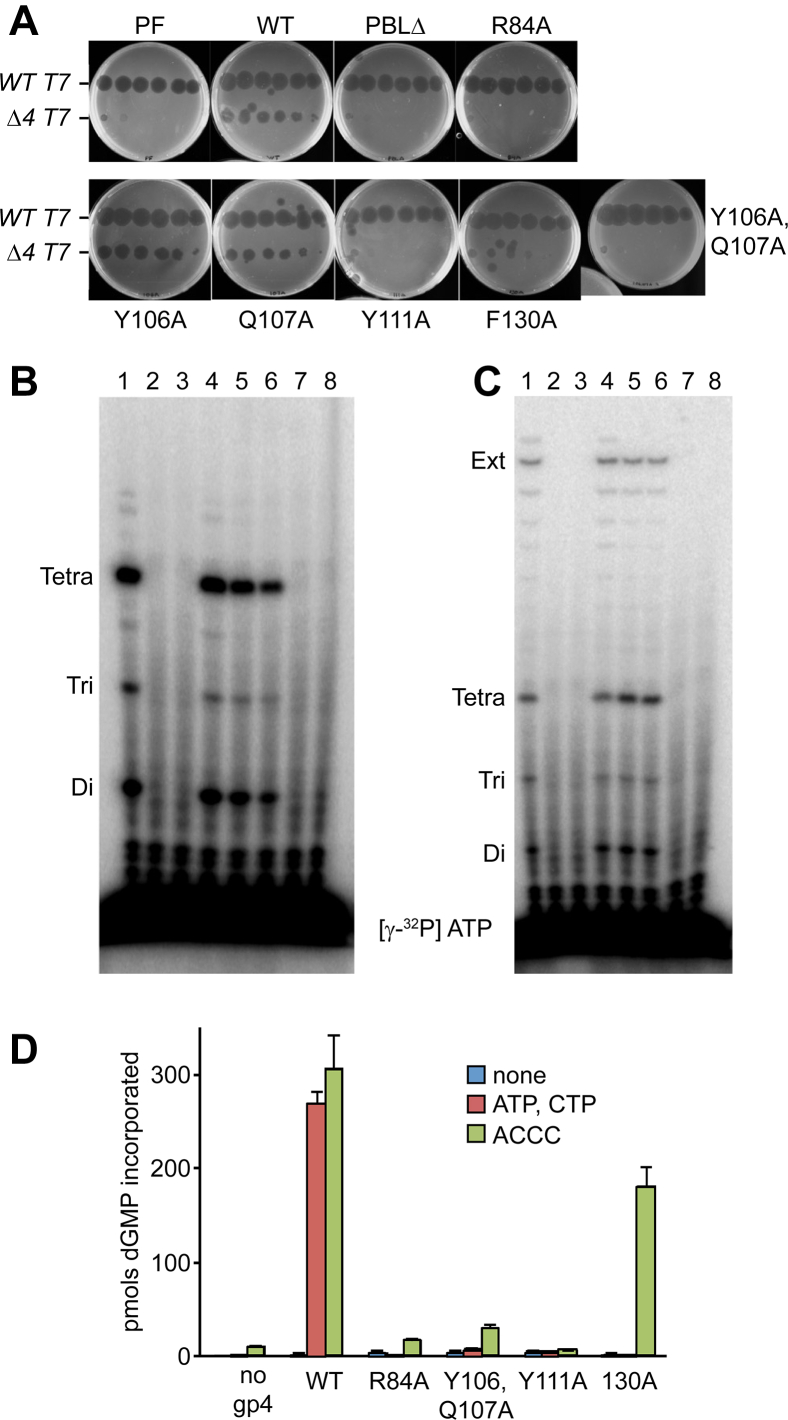
**Residues in the primer-binding region of gp4 are essential for phage growth and primer synthesis.***A*, genetic complementation assay. Serial tenfold dilutions of WT T7 or T7 Δ4 were spotted onto agar plates containing a lawn of *E. coli* DH5α transformed with plasmids encoding the indicated gp4 variants. Phage dilutions from left to right correspond to 10^−2^ to 10^−7^ dilutions of the parental phage stock. The ability of T7 to infect *E. coli* DH5α results in an area devoid of bacterial growth. Cells transformed with plasmids encoding WT gp4 and the T7 primase fragment (PF) served as positive and negative complementation controls, respectively. ΔPBL is a Δ101 to 111 gene 4 allele. *B*, *de novo* primer synthesis using an oligonucleotide template. WT gp4 or the indicated gp4 variants (0.1 μM hexamer concentration) (lane 1-WT gp4, lane 2 Δ101–111, lane 3 R84A, lane 4 Y106A, lane 5 YA, lane 6 Y106, Q107A, lane 7 Y111A, and lane 8 F130A) were incubated in reaction buffer in the presence of a ssDNA template containing a primase recognition sequence, 0.3 mM dNTPs, 0.1 mM [γ-^32^P] ATP (0.2 mCi/ml), and 0.1 mM CTP for 5 min at 25 °C. Reactions were quenched with formamide loading dye, products separated by denaturing gel electrophoresis, and visualized by phosphor imager. Products are labeled to the left of the gel image. *C*, RNA-dependent DNA synthesis using an oligonucleotide template. WT gp4 or the indicated gp4 variants (0.1 μM hexamer) were incubated in reaction buffer in the presence of a ssDNA template containing a primase recognition sequence, 0.3 mM dNTPs, 0.1 mM [γ-^32^P] ATP (0.2 mCi/ml) and 0.1 mM CTP, and 0.4 μM exonuclease-deficient T7 DNA polymerase for 5 min at 25 °C. lane 1 WT gp4, 2-Δ101 to 111, 3-R84A, 4-Y106A, 5- Q107A, 6-Y106, Q107A, 7-Y111A, and 8-F130A. Reactions were quenched with formamide loading dye, products separated by denaturing gel electrophoresis, and visualized by phosphor imager. Products are labeled to the left of the gel image. *D*, RNA-dependent DNA synthesis using M13 ssDNA as template. In this assay DNA synthesis catalyzed by T7 DNA polymerase on M13 ssDNA using primers synthesized by gp4 or preformed RNA primers is measured. WT gp4 and gp4 variants (50 nM hexamer concentration) were incubated in reaction buffer in the presence of 10 nM M13 ssDNA, 0.3 mM dNTPs (with 0.2 mCi/ml [α-^32^P] dGTP, 0.1 mM ATP, 0.1 mM CTP (or 0.1 mM preformed ACCC primer), and 20 nM T7 DNA polymerase for 10 min at 25 °C. Reactions were stopped with EDTA, spotted onto DE81 filters, and dGMP incorporation was measured using a liquid scintillation counter. Data represent the mean and SD of dGMP incorporation from at least three independent experiments. “None” indicates control reactions lacking ribonucleoside triphosphates or preformed RNA primer. dGMP, deoxy-GMP; dNTP, deoxy-NTP.

A genetic complementation assay was used to determine if residues described in the previous section are important for the function of the gp4 in supporting T7 growth. This genetic assay depends on the inability of a T7 phage (T7 Δ4) bearing a deletion in gene 4, which encodes for the primase-helicase gp4, to infect *E. coli*. Growth of T7 Δ4 is dependent on the presence of a functional gene 4 allele provided in *trans* on a plasmid. Specifically, we tested the role of three residues within the primer-binding region, Y111, Y106, and Q107, as well as two residues, F130 and R84, that line the RNA polymerase active site. R84 is highly conserved among DnaG homologs and lies in close proximity to the primase active site (32). Deletion of amino acid residues 101 to 111 of the primer-binding loop (ΔPBL, or Δ101–111) is lethal, as is the alanine substitutions of R84, Y111, and F130. Replacement of Y106 or Q107 to alanine results in normal growth, but an allele bearing a double substitution of Y106 and Q107 to alanine is unable to complement growth of T7 Δ4 (Fig. 3*A*).

### Biochemical activities of variant primase-helicases

The gp4 variants that could not support T7 growth were purified, and the biochemical activities of the helicase and primase were examined.

#### Primer synthesis

WT gp4 catalyzes the synthesis of diribonucleotides, triribonucleotides, and tetraribonucleotides in a template-mediated reaction (33). In an assay using a ssDNA template containing the primase recognition sequence 3′-CTGGT-5, CTP, and [γ-^32^P] ATP, WT gp4 catalyzes the synthesis of 5′-pppAC-3′, 5′-pppACC-3′, and 5′-pppACCA-3′, in which the terminal 5′ phosphate is radioactively labeled (Fig. 3*B* lane 1). Deletion of residues 101 to 111 and single alanine substitutions at positions 84, 111, and 130 abolishes primer synthesis (Fig. 3*B* lanes 3, 7, and 8). No defect was found in primer synthesis activity of gp4 variants bearing alanine instead of Y106 or Q107, (Fig. 3*B* lanes 4 and 5). This finding is consistent with the ability of these variants to complement the growth of T7 Δ4 phage. Surprisingly, the double alanine variant Y106A/Q107A that is lethal to T7 phage growth was able to synthesize primers at levels comparable to the WT enzyme (Fig. 3*B* lane 6).

#### RNA-primed DNA synthesis

T7 DNA polymerase interacts with gp4 and extends tetraribonucleotide primers synthesized by gp4 (13, 22). In order to determine if T7 DNA polymerase *via* interactions with gp4 could restore primer synthesis to the variant gp4, we included T7 DNA polymerase in the reactions described for primer synthesis. In this assay, T7 DNA polymerase extends tetraribonucleotides synthesized by gp4 (Fig. 3*C* lane 1). The presence of T7 DNA polymerase does not stimulate the synthesis of primers by gp4; variants of gp4 unable to synthesize RNA primers were still inactive in the presence of T7 DNA polymerase. (Fig. 3*C* lanes 2–3 and 7–8) Comparable levels of extension of primers synthesized by gp4 variants bearing single or double alanine substitutions in Y106 and Y111 werer observed (Fig. 3*C* lanes 4–6).

Gp4 can also transfer preformed tetraribonucleotides to T7 DNA polymerase to provide primers for extension by T7 DNA polymerase, enabling the latter to use these short RNAs as primers. In the experiment shown in Fig. 3*D*, T7 DNA polymerase catalyzed DNA synthesis in the presence of WT gp4, M13 ssDNA, and the preformed ribonucleotide primer ACCC. However, no DNA synthesis is observed for Δ101 to 111, R84A, or Y111A variants. Contrary to the results observed in the experiment described in Fig. 3*C*, the Y106A/Q107A variants supported less than 10% of the WT level of DNA synthesis. This observation raises the possibility that mutation of Y106A/Q107A of the primase domain leads to suboptimal primer delivery using this template. We also observed a separation of function of primer synthesis and delivery activities in gp4 F130A. While gp4 F130A is unable to synthesize an RNA primer, it functions efficiently in their delivery (Fig. 3, *A* and *D*). These results suggest that different, but partially overlapping, sets of residues in the primase domain mediate primer synthesis and delivery (See Discussion).

#### Strand-displacement DNA synthesis

**Figure 4:**
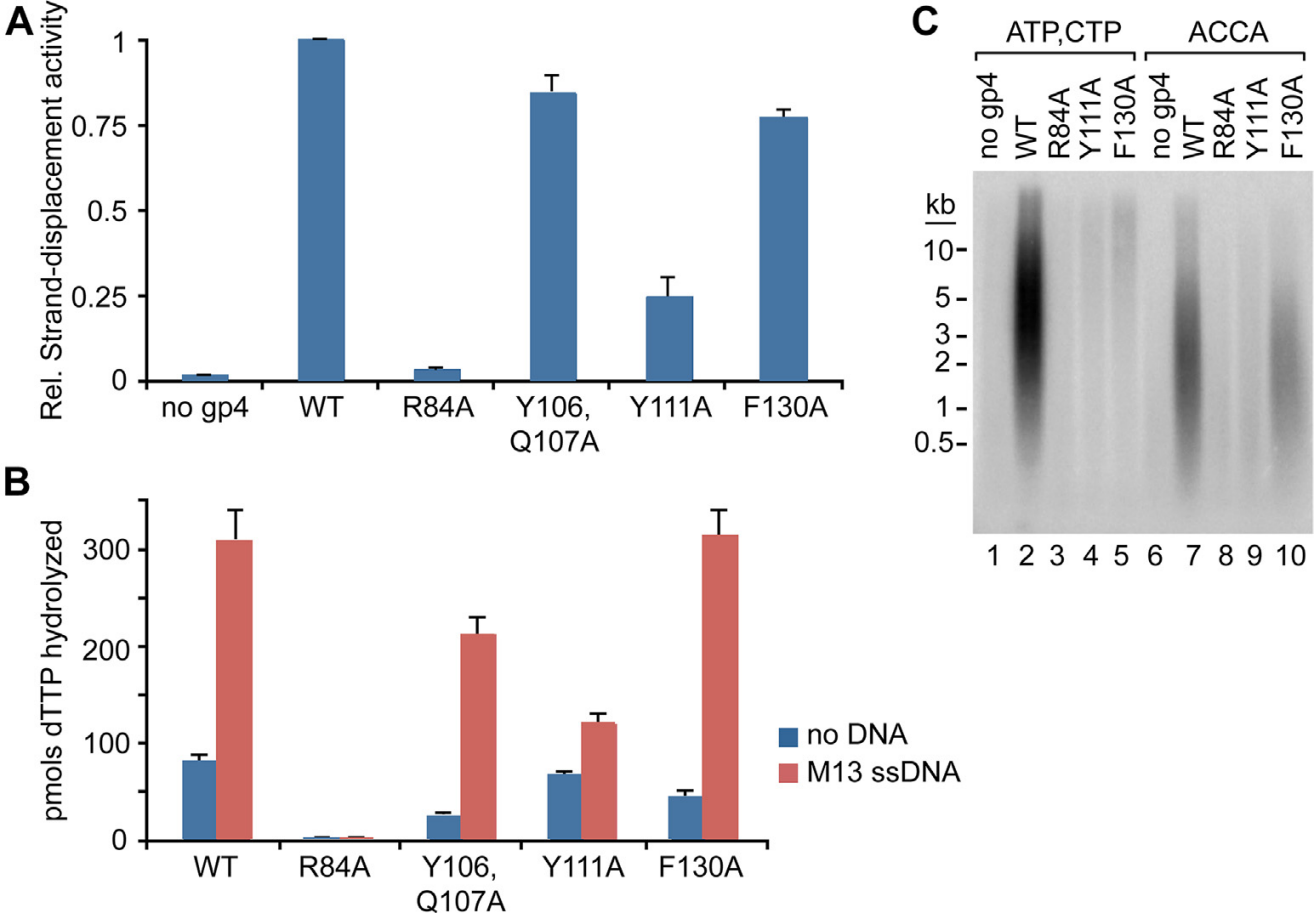
**Residues in the primer-binding region of gp4 affect the enzymatic activities of the gp4 helicase domain**. *A*, strand-displacement DNA synthesis. gp4 enables T7 DNA polymerase to catalyze DNA synthesis using dsDNA as template by unwinding dsDNA using the energy from dTTP hydrolysis and thus providing a ss template. M13 dsDNA (10 nM) containing a replication fork was incubated in the presence of 15 nM (hexamer concentration) of WT or the indicated gp4 variants and 20 nM T7 DNA polymerase in reaction buffer containing 0.3 mM dNTPs (with 0.2 mCi/ml [α-^32^P] dTTP) for 10 min at 25 °C. Reactions were stopped with EDTA, spotted onto DE81 filters, and incorporation of dTMP was measured with a liquid scintillation counter. Data represent the mean and SD of dTMP incorporation normalized to dTMP incorporated in reactions with the WT gp4, from at least three independent experiments. *B*, dTTP hydrolysis; gp4 catalyzes the hydrolysis of dTTP to dTDP and P_i_ in a reaction that is greatly increased by the presence of ssDNA on which it translocates. Reactions contained 50 nM of the indicated gp4 hexamer in reaction buffer and 1 mM [α-^32^P] dTTP (0.1 mCi/ml) in the absence or presence of 10 nM M13 ssDNA. Hydrolysis by gp4 was quenched by the addition of EDTA, products were separated from substrate by thin-layer chromatography, and radioactive signal in [^32^P] dTDP was visualized by phosphor imager. Data represent the mean and SD of dTDP produced from at least three independent experiments. (gp4 indicated on *x* axis) *C*, coordinated DNA synthesis: T7 DNA polymerase and gp4 mediate coordinated leading and lagging strand DNA synthesis on a synthetic minicircle DNA. The sequence bias of the minicircle DNA substrate allows for monitoring of synthesis of the leading and lagging strands by measuring the incorporation of dGMP or dCMP, respectively. Reactions (see “Experimental procedures”) contained WT gp4 or each of the indicated gp4 variants (1-No gp4, 2-WT gp4, 3-R84A, 4-Y111A, 5-F130A), using either nucleoside triphosphates (0.3 mM ATP and CTP) for primer synthesis or a preformed RNA primer (0.3 mM ACCA) for delivery, and 0.2 mCi/ml [α-^32^P] dCTP to exclusively label lagging-strand products. After 5 min at 30 °C, reactions were quenched with EDTA and products were separated on a 0.8% alkaline agarose gel and visualized by phosphor imager. Size markers are indicated to the *left* of the gel image. dGMP, deoxy-GMP; dNTP, deoxy-NTP; dTDP, deoxy-TDP; dTTP, deoxy-TTP.

The helicase activity of gp4 unwinds dsDNA and provides the single-stranded template required for DNA synthesis by T7 DNA polymerase, a process that occurs during leading strand DNA synthesis. We examined gp4 variants for their ability to support leading strand DNA synthesis using M13 dsDNA as template for strand-displacement synthesis. In this assay M13 dsDNA containing a preformed replication fork is incubated with T7 DNA polymerase and gp4 and the amount of DNA synthesis is measured; controls consisted of no gp4 or WT gp4 (see inset to Fig. 4*A*). All gp4 variants, with the exception of those containing either the R84A or Y111A mutation, supported strand-displacement synthesis, and at least 75% of the level of incorporation was observed for WT gp4 (Fig. 4*A*). No strand displacement synthesis is observed with gp4 R84A and only 20% of that obtained with WT gp4 is found with gp4 Y111A.

#### dTTPase activity

The defects in strand-displacement synthesis observed with gp4-R84A and gp4-Y111A suggest that helicase activity is severely compromised despite the location of these mutations in the primase domain. Gp4 couples the hydrolysis of nucleoside triphosphates to translocation on ssDNA and to unwinding dsDNA, using deoxy-TTP (dTTP) as its preferred substrate (34). Therefore, a sensitive assay for the ability of gp4 to translocate on ssDNA is to examine the dTTPase activity of gp4 in the presence of ssDNA (see inset to Fig. 4*B*). Gp4 also catalyzes dTTP hydrolysis in the absence of ssDNA but at a considerably lower rate (Fig. 4*B*). Gp4 R84A does not catalyze the hydrolysis of dTTP either in the presence or absence of DNA. The other variants displayed some level of dTTPase activity in the absence of ssDNA and were all stimulated by the presence of ssDNA with gp4 F130A resembling WT gp4.

#### Lagging-strand synthesis during coordinated DNA synthesis

The enzymatic activities of the primase-helicase gp4 are essential to the function of the T7 replisome. Gp4 unwinds dsDNA ahead of the replication fork and provides primers for the discontinuous synthesis of the lagging strand (5, 35). It interacts with the leading and lagging strand DNA polymerases, securing them in position for DNA synthesis (6, 7). It seems unlikely that the gp4-R84A and gp4-Y111A variants could provide primers and their transfer to the lagging strand DNA polymerase, since the individual reactions of each process is defective. Nevertheless, the possibility remained that these variants might display retain show activity as a consequence of the multiple interactions during coordinated DNA synthesis. We used a minicircle DNA to measure the effect of alanine substitution of R84, Y111, and F130 on lagging-strand synthesis under conditions of coordinated DNA synthesis.

The sequence composition of the minicircle template permits monitoring synthesis of each strand in an exclusive manner by measuring incorporation of either deoxy-GMP (dGMP) or deoxy-CMP, for leading and lagging strands, respectively. Under conditions where DNA synthesis is dependent on the ability of gp4 to synthesize and deliver RNA primers from ATP and CTP substrates (*de novo* primer synthesis), lagging-strand synthesis products were detectable only in the presence of WT gp4 (Fig. 4*C*, lane 2). In assays where we supplied a preformed RNA primer instead of ATP and CTP, WT gp4 and gp4-F130A can support lagging-strand synthesis (lanes 7 and 10). No significant accumulation of lagging-strand products was detectable with gp4-R84A or gp4-Y111A (lanes 3–4 and 8–9).

### Synthesis by the primase domain of gp4 is not affected by the R84A mutation

**Figure 5:**
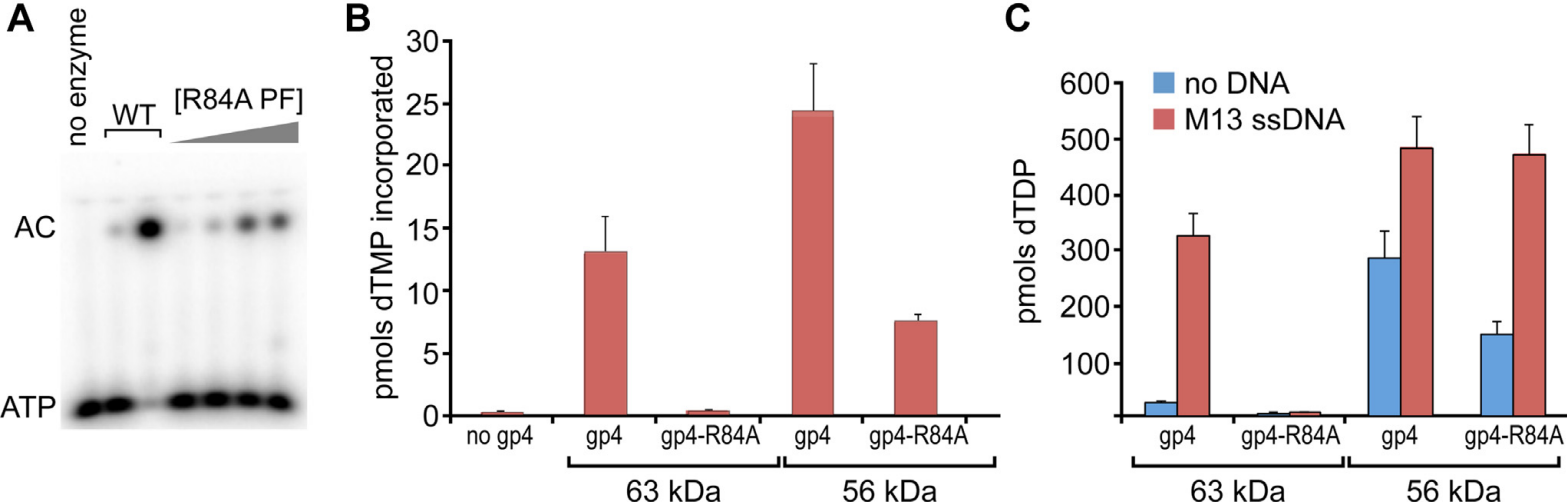
**Effect of R84A mutation on the activities of the helicase and primase domains.***A*, diribonucleotide synthesis catalyzed by WT and R84A T7 primase fragment (residues 1–257 of gp4). 0.1 and 10 μM T7 WT primase fragment or 0.01 to 10 μM primase fragment containing the R84A mutation were assayed using a DNA template containing the recognition sequence 5′-GTC-3’. The reaction contained ssDNA template, 0.1 mM ATP, and 0.1 mM [α-^32^P] CTP (0.2 mCi/ml) for 10 min at 25 °C. Reactions were quenched with formamide loading dye, products separated by denaturing gel electrophoresis, and visualized with a phosphor imager. The identities of the products are indicated to the *left* of the gel image. *B*, strand-displacement DNA synthesis facilitated by 56 kDa gp4 and 63 kDa variants, each bearing the R84A mutation. The reactions contained 10 nM M13 dsDNA containing a replication fork. Fifteen nanomole gp4 (hexamer concentration) and 20 nM T7 DNA polymerase in reaction buffer containing 0.3 mM dNTPs (with 0.2 mCi/ml [α-^32^P] dGTP. After incubation for 10 min at 25 °C, the reactions were stopped with EDTA, spotted onto DE81 filters, and dGMP incorporation was measured on a liquid scintillation counter. Data represent the mean and SD of dGMP incorporated from at least three independent experiments. *C*, dTTPase activity of the 56 and 63 kDa forms of gp4 bearing an R84A substitution. Reactions contained either 56 or 63 kDa gp4 (50 nM hexamer), reaction buffer, and 1 mM [α-^32^P] dTTP (0.1 mCi/ml) in the absence or presence of 10 nM M13 ssDNA. Hydrolysis by gp4 was quenched by the addition of EDTA, products were separated from substrate by thin-layer chromatography, and radioactive signal was visualized by a phosphor imager. Data represent the mean and SD of dTDP produced from at least three independent experiments. dGMP, deoxy-GMP; dGTP, deoxy-GTP; dNTP, deoxy-NTP; dTDP, deoxy-TDP; dTTP, deoxy-TTP.

The primase domain of gp4 when not linked to the helicase domain retains primase activity (36). However, the activity of the primase fragment is reduced, particularly on large DNA templates, due to its inability to translocate on ssDNA to access primase recognition sites, an activity conferred by the helicase domain (36, 37, 38). The R84A mutation does not inactivate primer synthesis of the purified primase fragment (Fig. 5*A*, compare lanes 2 and 3 with lanes 4–7), in contrast to the inactivation observed with the full-length gp4 R84A described in a previous section (Fig. 3, *B* and *C*, lanes 3).

*In vivo* expression of gene 4 results in the production of equimolar amounts of a full-length 63 kDa form and a 56 kDa form (39). The 56 kDa form of gp4 lacks the ZBD in the primase domain, owing to the usage of an alternative initiation at codon position 64 in the gene 4 gene. This results in a polypeptide composed of residues 64 to 566 of gp4 that lacks primer synthesis activity but retains full helicase activity (40, 41). A 56 kDa gp4 variant bearing an alanine substitution at the arginine corresponding to R84 in the 63 kDa isoform (56 kDa gp4-R84A, for simplicity) retains helicase activity, as shown by its high ssDNA-dependent dTTPase activity and its ability to support strand-displacement DNA synthesis at levels 70% of WT (63 kDa) gp4A (Fig. 5, *B* and *C*). Thus, unlike the 63 kDa form, an alanine substitution of R84 in the 56 kDa form of gp4 does not disrupt helicase activities mediated by DNA binding. This result suggests that in the absence of the ZBD, R84 might not play a major role in loading of gp4 onto ssDNA.

### Primase domain mutations decrease DNA-binding affinity but do not alter the oligomerization equilibrium of gp4

**Figure 6:**
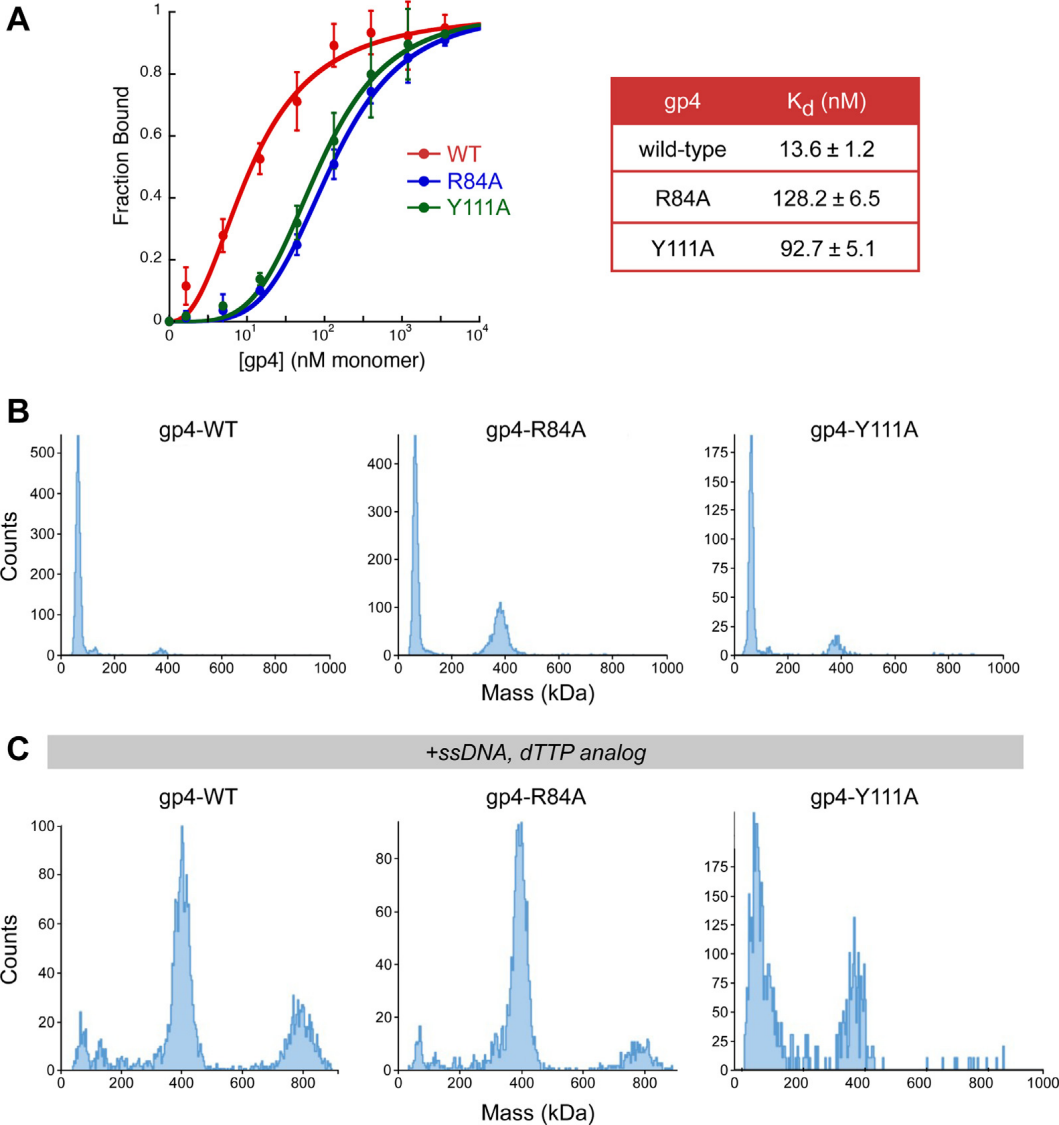
**Effect of substitution of alanine for R84 and Y111 in gp4 on ssDNA affinity and oligomerization**. *A*, affinity of gp4 for ssDNA. A double-filter DNA-binding assay was used to measure the affinity of WT gp4 and g4-R84A and gp4-Y111A to ssDNA. Binding isotherms were measured in a reaction containing ^32^P 5′-end labeled 50-nt ssDNA (1 nM) with increasing concentrations (1.6 nM-3.6 μM) of WT gp4 (*red*), gp4-R84A (*blue*), or gp4-Y111A (*green*) in the presence of 1 mM [β,γ-CH_2_] dTTP. Binding reactions were filtered through nitrocellulose and Zeta probe membranes, and the radioactivity on each membrane was measured. The fraction ssDNA bound as a function of gp4 concentration is shown. Data represent the mean and SD of fraction ^32^P-ssDNA bound as a function of gp4 concentration from at least three independent experiments. *B*, mass histogram of WT gp4 (*left*), gp4-R84A (*middle*), and gp4-Y111A (*right*) in the absence of ligand. Mass photometry measurements were carried out on samples containing 8.7 nM gp4 hexamer in 40 mM Tris–HCl, pH 7.5, 150 mM KCl, and 10 mM MgCl_2_. *C*, oligomerization of gp4. Mass histogram of WT gp4 (*left*), gp4-R84A (*middle*), and gp4-Y111A (*right*) were determined as in (*B*) after incubation with ssDNA and [β,γ-CH_2_] dTTP. dTTP, deoxy-TTP.

We compared the ability of WT, gp4-R84A, and gp4-Y111A to bind ssDNA using a nitrocellulose filter–binding assay. WT gp4 binds to ssDNA in the presence of nonhydrolyzable dTTP with an affinity of 14 nM (Fig. 6*A*). Under the same conditions, R84A and Y111A gp4 variants bind to ssDNA with significantly lower affinity than does WT gp4 (Fig. 6*A*). Gp4-R84A binds ssDNA with an approximately ninefold lower affinity, with a *K*_d_ of 128 nM. Similarly, Y111A gp4 binds ssDNA with a *K*_d_ of 93 nM, representing a, approximately sevenfold decrease in binding affinity.

Since gp4 binds to ssDNA as a hexamer, one explanation for decreased binding of the variants is the inability to oligomerize. The molecular masses of gp4 proteins were determined by mass photometry (38) in the absence of any ligand and in the presence of a combination of a nonhydrolyzable dTTP analog and ssDNA. In the absence of ssDNA, approximately 90 % of WT gp4 exhibits a mass of ∼60 kDa, consistent with a gp4 monomer (Fig. 6*B* and supporting information). Similarly, in the absence of DNA, gp4-R84A and gp4-Y111A exhibited a particle molecular mass profile consistent with a gp4 monomer (Fig. 6*B*). However, other species of higher molecular mass are also detectable, particularly for gp4-R84A, where ∼40% is found as a ∼400 kDa particle (Fig. 6*B* and supporting information).

The presence of ssDNA and nonhydrolyzable dTTP resulted in an increase in the molecular mass of WT gp4 to 400 to 440 kDa consistent with the molecular mass of a gp4 hexamer (Fig. 6*C*). Intermediate oligomers larger than the monomer but smaller than the hexamer are also present. These intermediates have mass differences that correspond approximately to the mass of a gp4 monomer. A heptameric form, previously described (16), was not discernable, but we cannot exclude the possibility that the measurements do not have the resolution to distinguish between a hexamer and a heptamer. We also observed particles with masses of ∼800 kDa, suggesting that two gp4 hexamers might reside on a single ssDNA molecule. The profile of gp-R86A closely resembles that of WT gp4 while only 26% of gp4-Y111A is converted to a hexameric species by the presence of ssDNA and nonhydrolyzable dTTP. In summary, both gp4-R84A and gp4-Y111A, have decreased affinity for ssDNA and exist in similar, but not identical, oligomeric distributions as WT gp4.

## Discussion

The multiple protein and nucleic acid interactions that occur at a replication fork coordinate the numerous reactions that lead to coordinated leading and lagging strand DNA synthesis. One of the more complex reactions is the synthesis of RNA primers by the DNA primase and their transfer to the lagging strand DNA polymerase, an event that also involves the DNA helicase, which interacts with the DNA, the primase, and the DNA polymerase. In order to identify interacting residues during the RNA priming event, we used formaldehyde to form zero-length crosslinks between protein and biotinylated nucleic acids during the course of primer synthesis and their transfer to T7 DNA polymerase. Of the multiple interactions identified, a peptide region in the primase domain linked to the RNA primer was of particular interest. This region consisted of residues 101 to 111 located close to primase motif III. This region is in close proximity to two essential lysines at positions 122 and 128, located near the N-terminal basic subdomain (12, 42). Gene 4 alleles bearing alanine substitutions at position 111 or doubly substituted at positions 106 and 107 within the primer-binding region of gp4 were unable to rescue the growth of phage lacking gene 4. In addition, two residues in close proximity to the primer-binding region, R84 and F130 were also found to be essential for phage growth. R84 is located at a distance of 3.3 Å to the Mg^2+^ in the RPD, suggesting a role in the metal-mediated catalytic step (12, 43). The aromatic side chains of Y111 and F130 are located on the same face of the protein and could be involved in stabilizing the primer/template through base-stacking interactions. Interestingly, two essential lysine residues (K122 and K128) are located in the vicinity of the aromatic side chains, possibly creating a nucleic acid–binding tract between the N-terminal beta-strand rich subdomain and the TOPRIM domain (42).

Biochemical analysis of purified gp4 containing mutations of residue identified *via* our affinity/mass spectrometry approach revealed multiple defects in helicase and primase function. Of particular interest are gp4 in which either R84 or Y111 are replaced with alanine. Although these altered proteins form oligomers including the functional hexamer, they have serious defects in binding to ssDNA. Not surprisingly, these altered gp4 do not hydrolyze dTTP in the presence of ssDNA and are unable to mediate strand displacement synthesis. Interestingly, both proteins are also defective in template-directed primase activity, although the primase domain, uncoupled from the helicase domain, does have primase activity.

Primer delivery can be uncoupled from primer synthesis. Mutation of residues involved in primer synthesis often results in gp4 that are still capable of primer delivery (42, 44). In line with this, we found that a F130A substitution makes gp4 unable to catalyze the synthesis of RNA primers from ribonucleoside triphosphate substrates, but this mutant still delivers preformed RNA primers to T7 DNA polymerase. We also found that a double alanine mutant at position 106 and 107 results in a gp4 that synthesizes RNA primers but is unable to deliver them to DNA polymerase. These results suggest that primer synthesis and delivery are processes facilitated by separate regions of the primase domain and/or through different conformational states (13, 22, 31, 42, 44).

What is the molecular basis for the inability of gp4 A84R and A111Y to bind to ssDNA? They both apparently form hexamers as well as WT gp4 so the defect would appear to be the ability to load onto the ssDNA. Analysis of the oligomeric state of gp4 by mass photometry leads us to reconsider previous models for the oligomeric behavior of gp4 in solution. In the absence of ligands, we find that gp4 exists in a monomeric state. Binding of dTTP and ssDNA leads to the formation of an oligomer with a size corresponding to a hexamer. The number of intermediates and the difference in mass between intermediates strongly suggest that formation of a gp4 hexamer is the result of a sequential and ordered assembly of monomers.

The oligomeric state of gp4 is essential for its role in DNA replication. Hexamers are the molecular species that bind ssDNA. This oligomeric arrangement forms the nucleotide-binding pocket between interfaces of adjacent helicase subunits, enables translocation of the helicase domain along ssDNA, and allows intersubunit communication through interactions in *trans* (14, 45, 46, 47, 48). Coordinated cycles of association and dissociation between subunits of the hexamer and ssDNA are responsible for translocation of gp4 along ssDNA (15, 49). The flexible linker region between the gp4 primase and helicase domain is essential for the formation of active hexamers (50, 51). This suggests that the interdomain and intersubunit communication of nucleotide- and DNA-binding states is critical for the function of gp4 at the replication fork (48, 51).

The stabilization of hexameric gp4 could come about through the more favorable release of binding energy afforded by the increased number and range of interactions that occur between primase and helicase domains of different subunits in the hexameric over the monomeric conformation. The deleterious effect of primase domain mutations we observe when we altered the identity of R84 and Y111 suggests that these residues are involved in the interdomain interactions that lead to the formation of active hexameric species.

In summary, we have identified a region in the primase domain of gp4 that is involved in synthesis of RNA primers and their hand off to DNA polymerase. Genetic and biochemical analyses of gp4 variants bearing mutations in residues within the primer-binding loop region suggest that communication between the primase and helicase domains of gp4 is essential for activity. Finally, our results suggest that gp4 hexamers are formed through an assembly pathway requiring the sequential addition of monomeric subunits.

## Experimental procedures

### Construction of plasmids and protein purification

Plasmids encoding gp5, exonuclease-deficient gp5 (D5A and D65A), the T7 ssDNA-binding protein, gp2.5, and Trx, have been described previously, and these proteins were purified as described (27, 52, 53, 54). Plasmids encoding variant gene 4 alleles used in this study were constructed by site-directed mutagenesis using pET28-gp4A as template.

#### Expression and purification of gp4

*E. coli* BL21 (DE3) transformed with pET28-gp4 constructs were grown in terrific broth containing 50 μg/ml kanamycin, 1.5 mM MgSO4, and 0.1 mM ZnCl_2_ at 37 °C. After reaching an *A*_600_ of 2, isopropyl thiogalactopyranoside was added to a final concentration of 1 mM, the shaker temperature was lowered to 16 °C, and growth was continued for 16 h. Gp4 proteins were purified by immobilized metal–affinity chromatography (TALON, Takara) followed by anion-exchange chromatography (DEAE-FF, Cytiva). Protein concentrations were determined using the Bradford reagent and UV measurements (55). All proteins were greater than 95% pure as judged by SDS-PAGE and Coomassie staining.

### Genetic complementation assay

*E. coli* DH5α were transformed with plasmids expressing WT or variant T7 gene 4 constructs, plated onto LB agar plates containing 50 μg/ml kanamycin, and grown overnight at 37 °C. Transformed cells were grown in LB to exponential phase. Three hundred microliter of bacterial culture was mixed with 3 ml of soft agar (LB broth + 0.7% agar) and the mixture was poured onto LB agar/kanamycin plates. Aliquots of serially diluted WT or gene 4 deletion (Δ4) T7 phage were spotted onto the plates to determine phage titer.

### Identification of nucleic acid–interacting protein regions

The T7 priming complex was assembled by incubating 1.5 μM gp4 hexamer and 5 μM gp5/trx with either 3 μM template (5′-CAGTGACGGGTCGTTTATCGTCGGCA-3′, IDT) plus 3 μM biotinylated RNA primer (5′-Biotin-ACCC-3′, Dharmacon) or 3 μM biotinylated template (5′-CAGTGACGGGTCGTTTATCGTCGGCA-Biotin-3′, IDT) plus 3 μM RNA primer (5′-ACCC-3′) in the presence of 0.3 mM deoxy-GTP (dGTP), 0.3 mM dideoxy-TTP, and 10 mM deoxy-CTP in a buffer containing 40 mM Hepes–KOH, pH 7.5, 50 mM potassium glutamate, 10 mM MgCl_2_, and 5 mM DTT for 30 min at 25 °C in a 100 μl reaction.

Formaldehyde was added to the aforementioned reaction mixture at a concentration of 0.1% and incubated at 25 °C for 5 min. Crosslinking was quenched by addition of Tris, pH 8.0 to 0.2 M. Sequencing grade trypsin (Pierce) was added at a final concentration of 5 ng/μl in 50 mM bicarbonate buffer and the samples were incubated at 37 °C overnight.

Samples were incubated with 1 mg of streptavidin-coupled magnetic beads (Dynabeads, Invitrogen), and unbound peptides were extensively washed with 20 mM Hepes (pH 7.5), 1 M NaCl, 1 mM EDTA, and 1 mM DTT. The nucleic acid–peptide conjugates were reversed by incubating the samples at 70 °C for 1 h. The supernatant containing the peptides was desalted using Ziptips (Millipore) as described (56). Bound peptides were eluted in 2.5 μl of 70% acetonitrile/0.1% TFA and were analyzed by MALDI-TOF mass spectrometry (Taplin Mass Spectrometry Facility – Harvard Medical School).

### Enzymatic assays

#### Primer synthesis *de novo*

Assays consisted of 0.1 μM gp4 hexamer, 0.1 μM ssDNA template (5′-CAGTGACGGGTCGTTTATCGTCGGCA-3′, IDT), 0.1 mM ATP, 0.1 mM CTP, 0.2 mCi/ml [γ-^32^P] ATP, and 0.3 mM dNTPs in 40 mM Tris–HCl, pH 7.5, 50 mM potassium glutamate, 10 mM MgCl_2_, and 5 mM DTT. Samples were incubated at 25 °C for the time indicated and quenched with an equal volume of formamide loading dye (93% (v/v) formamide, 50 mM EDTA, 0.01% xylene cyanol, and 0.01% bromophenol blue), followed by denaturing gel electrophoresis and phosphor imaging.

#### RNA-primed DNA synthesis

Assays using an ssDNA oligonucleotide template were identical to those used for *de novo* primer synthesis with the addition of 0.4 μM exo^-^ gp5/Trx. When a preformed tetraribonucleotide primer was used instead of ATP and CTP, we included 0.2 mCi/ml [α-^32^P] dGTP as a label.

Assays that used M13 ssDNA as template contained 10 nM M13 ssDNA, 0.3 mM dNTPs, 0.2 mCi/ml [α-^32^P] dGTP, 20 nM WT gp5/Trx, and 300 nM gp4 in 40 mM Tris–HCl, pH 7.5, 50 mM potassium glutamate, 10 mM MgCl_2_, and 5 mM DTT. Samples were incubated at 25 °C for 10 min and quenched by the addition of and equal volume of 50 mM EDTA. Samples were applied to DE81 filters, washed, and dGMP incorporation into DNA was quantified using a liquid scintillation counter.

#### Strand-displacement DNA synthesis

Strand-displacement DNA synthesis reactions contained 10 nM M13 dsDNA template, prepared as described (28), 0.6 mM dNTPs, 0.2 mCi/ml [α-^32^P] dGTP, 20 nM WT T7 DNA polymerase and 90 nM gp4 in 40 mM Tris–HCl, pH 7.5, 50 mM potassium glutamate, 10 mM MgCl_2_, and 5 mM DTT. After incubation at 37 °C for 15 min, the reactions were terminated by addition of an equal volume of 50 mM EDTA, and the amount of dGMP incorporation into DNA was quantified using a liquid scintillation counter.

#### dTTPase assay

The indicated concentrations of gp4 was incubated in 40 mM Tris–HCl, pH 7.5, 50 mM potassium glutamate, 10 mM MgCl_2_, 5 mM DTT, and 1 mM ^32^P-dTTP (0.1 mCi/ml, Perkin) for 10 min at 25 °C either in the presence or in the absence of 10 nM ssM13 DNA. Reactions were stopped by addition of EDTA to a final concentration of 50 mM, spotted on PEI-cellulose TLC plates, and developed with 1M formic acid/0.8 M LiCl. Hydrolysis products were quantified by phosphor imaging.

#### Coordinated DNA synthesis

The DNA minicircle with a replication fork was constructed as described (57). Coordinated DNA synthesis assays contained 10 nM minicircle DNA, 600 μM dNTPs 0.2 mCi/ml [α-^32^P] deoxy-CTP, 300 μM ATP and CTP (or alternatively 0.3 μM ACCA primer), 80 nM gp5/Trx, 60 nM gp4, and 3 μM gp2.5 in 40 mM Tris–HCl, pH 7.5, 50 mM potassium glutamate, 10 mM MgCl_2_, and 5 mM DTT. An equal volume of 50 mM EDTA was added after incubation at 30 °C for 5 min, and the samples were loaded onto an alkaline agarose gel. The gel was dried, and labeled DNA was detected by phosphor imager.

### DNA-binding assays

One nanomole 5′-^32^P labeled ssDNA (5′-ATGACCATGATTTCGACGTTTTTTTTTTTGGGGAT CCTCTAACCTGCGCA-3′, IDT) was titrated with purified gp4 and incubated in 40 mM Tris–HCl, pH 7.5, 50 mM potassium glutamate, 10 mM MgCl_2_, 5 mM DTT, and 1 mM [β,γ-CH_2_] dTTP for 30 min at 25 °C. After incubation, samples were filtered through a nitrocellulose membrane (Schleicher & Schuell) and a Zeta-Probe membrane (Bio-Rad) on a microfiltration apparatus (Bio-Rad). Bound and free ssDNA was quantified by phosphor imager.

### Mass photometry

The 8.7 nM (hexameric concentration) of WT gp4, gp4-R84A, or gp4-Y11A in 40 mM Tris–HCl, pH 7.5, 150 mM KCl, and 10 mM MgCl_2_ were incubated at 25 °C for 15 min in the absence of ligand or in the presence of 10 nM ssDNA (5′-ATGACCATGATTTCGACGTTTTTTTTTTTGGGGAT CCTCTAACCTGCGCA-3′, IDT) and 1 mM [β,γ-CH_2_] dTTP. After incubation, samples were loaded onto a coverslip with a silicone gasket an analyzed using a Refeyn OneMP instrument (Refeyn). Six thousand frames were recorded, and images were processed and analyzed using DiscoverMP (Refeyn).

## Data availability

All data presented are contained in the article and is available upon request from Alfredo J. Hernandez, Harvard Medical School, alfredo_hernandez@hms.harvard.edu.

## Supporting information

This article contains supporting information.

## Conflict of interest

The authors declare that they have no conflicts of interest with the contents of this article.
