# Supplementary material for: Residues located in the primase domain of the bacteriophage T7 primase-helicase are essential for loading the hexameric complex onto DNA
Source: J Biol Chem. 2022 Apr 30;298(6):101996. doi: 10.1016/j.jbc.2022.101996 (PMC9198812; doi:10.1016/j.jbc.2022.101996)
Supplement: Supporting information [file mmc1.pdf]

Supporting Information

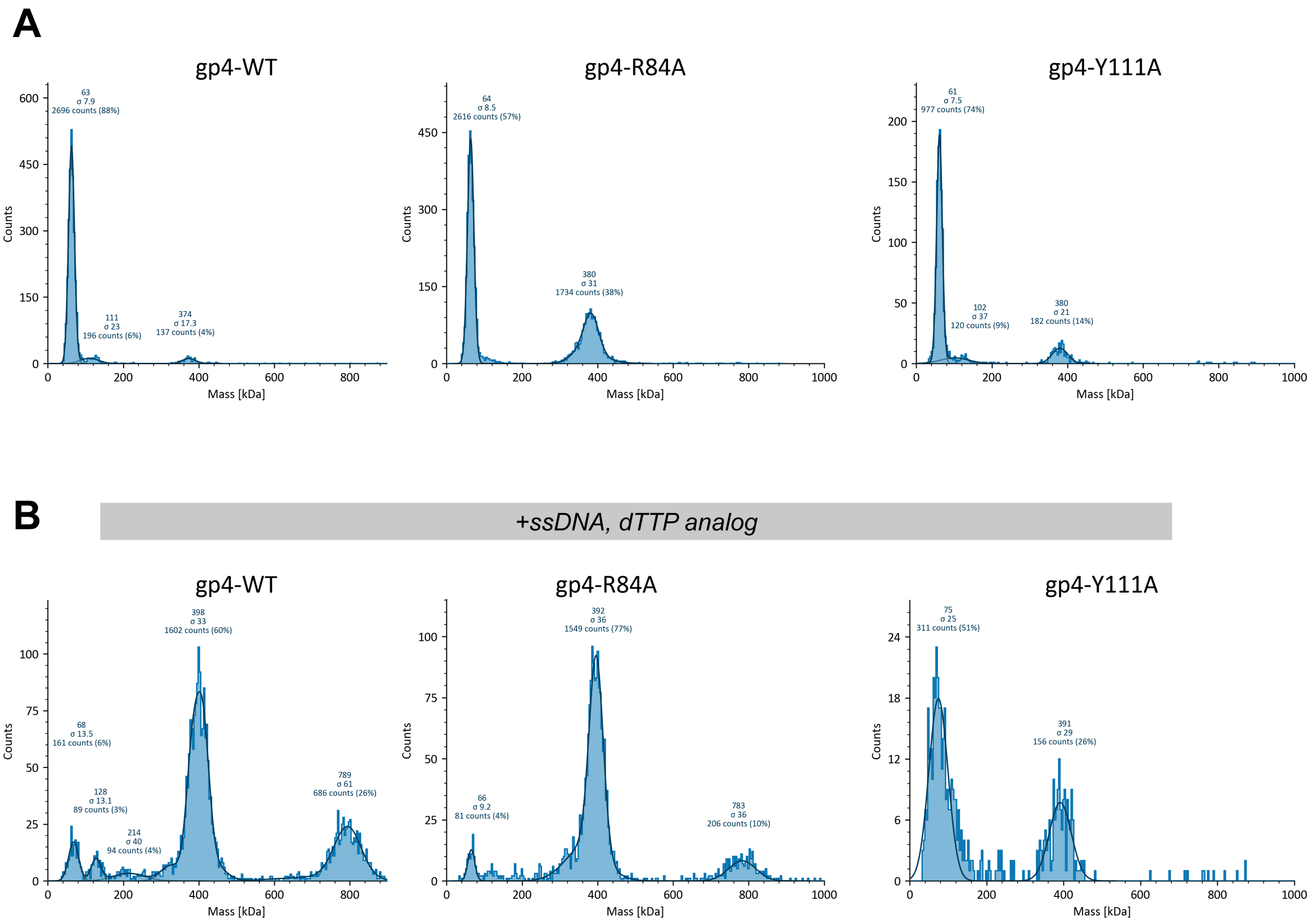

Masses of gp4 oligomers determined using DiscoverMP.

A, Molecular mass of oligomeric species in main text figure 6B as fit by DiscoveryMP software. Mass measurements of oligomers observed for wild-type gp4 (left), gp4-R84A (middle), and gp4-Y111A (right) in the absence of ligand (8.7 nM gp4 hexamer in 40 mM Tris-HCl, pH 7.5, 150 mM KCl, and 10 mM $MgCl_2$) are shown above the fitted curve.

B, Molecular mass of gp4 oligomers in main text figure 6C as fit by DiscoveryMP software. Mass fits are for wild-type gp4 (left), gp4-R84A (middle), and gp4-Y111A (right) were determined as in **A** after incubation with ssDNA and [β,γ-CH_2] dTTP. Oligomeric masses are shown above the fitted curve.
